# AUI&GIV: Recommendation with Asymmetric User Influence and Global Importance Value

**DOI:** 10.1371/journal.pone.0147944

**Published:** 2016-02-01

**Authors:** Zhi-Lin Zhao, Chang-Dong Wang, Jian-Huang Lai

**Affiliations:** 1 School of Data and Computer Science, Sun Yat-sen University, Guangzhou, China; 2 Guangdong Key Laboratory of Information Security Technology, Guangzhou, China; University of Amsterdam, NETHERLANDS

## Abstract

The user-based collaborative filtering (CF) algorithm is one of the most popular approaches for making recommendation. Despite its success, the traditional user-based CF algorithm suffers one serious problem that it only measures the influence between two users based on their symmetric similarities calculated by their consumption histories. It means that, for a pair of users, the influences on each other are the same, which however may not be true. Intuitively, an expert may have an impact on a novice user but a novice user may not affect an expert at all. Besides, each user may possess a global importance factor that affects his/her influence to the remaining users. To this end, in this paper, we propose an asymmetric user influence model to measure the directed influence between two users and adopt the PageRank algorithm to calculate the global importance value of each user. And then the directed influence values and the global importance values are integrated to deduce the final influence values between two users. Finally, we use the final influence values to improve the performance of the traditional user-based CF algorithm. Extensive experiments have been conducted, the results of which have confirmed that both the asymmetric user influence model and global importance value play key roles in improving recommendation accuracy, and hence the proposed method significantly outperforms the existing recommendation algorithms, in particular the user-based CF algorithm on the datasets of high rating density.

## Introduction

In the era of information explosion, it is difficult for us to select useful information before we make a decision among a large number of choices in a short time. The recommendation system becomes a necessity and is widely used in many e-commerce platforms like Amazon, Drugstore, Walmart, etc [1–10]. The goal of the recommendation system is to filter useful information from an enormous amount of information so that we can predict the rating that a user would give to an item and hence recommend items to the right users. Generally, recommendation algorithms can be classified into three different types [11], namely collaborative filtering algorithm [12–14], content-based algorithm [15] and hybrid recommendation algorithm [16].

Collaborative filtering is one of the most successful technologies in personalized recommendation and widely used in many websites [12, 17–26]. The collaborative filtering algorithm utilizes a large amount of users’ rating records to predict which items the target user will like. There are two kinds of collaborative filtering algorithms, one of which is user-based collaborative filtering [27] and the other is item-based collaborative filtering [28]. The user-based collaborative filtering algorithm computes the users’ similarities [29] according to the users’ rating records and recommend the items that the similar users have purchased to the target user. The most widely used similarities include cosine-based similarity, correlation-based similarity and adjust-cosine similarity, all of which are symmetric similarities [27, 29, 30]. On the contrary, the item-based collaborative filtering algorithm measures the similarities between items and recommends the items which are most similar to the items the target user has already bought to the target user.

Due to the fact that the user’s buying behavior is often affected by others, the user-based collaborative filtering (CF) algorithm has received a great amount of attention and many variants have been developed [27]. However, most of the existing user-based CF algorithms measure the influence between two users according to their symmetric similarities calculated from the consumption histories. It means that, for a pair of users, the influences on each other are the same, which however may not be true. Intuitively, an expert may have an impact on a novice user but a novice user may not affect an expert at all. Besides, each user may possess a global importance factor that affects his/her influence to the remaining users. Although in [31], both the asymmetric user similarity and the *implicit* global importance are used, the preference of users is not taken into account and the *implicit* global importance is not based on the asymmetric similarity. In fact, the preference of users would be different even if the items they have already purchased are the same and the explicit global importance value of each user would be calculated from the asymmetric similarity, both of which are considered in the proposed algorithm.

To address the above issues, this paper proposes a novel user-based collaborative filtering algorithm termed Asymmetric User Influence and Global Importance Values (AUI&GIV). An asymmetric user influence model is designed to measure the directed influence between two users and the PageRank algorithm is utilized to calculate the global importance value of each user. We define the positive items according to users’ rating records and use those positive items to measure the directed influence between users. Afterwards, the PageRank algorithm is used to calculate all users’ global influence values by the directed influence obtained in the previous step. And then the directed influence values and the global importance values are combined to deduce the final influence values between two users. Finally, the final influence values are used to improve the performance of the traditional user-based CF algorithm.

In our experiments, we compare our recommendation algorithm with seven recommendation algorithms on four widely-tested datasets like MovieLens, Jesters, EachMovie and Netflix. Experimental results show that our algorithm can achieve a better performance than the existing recommendation algorithms. Significance tests also show that the proposed algorithm can significantly improve the performance of the traditional user-based CF algorithm on datasets of high rating density.

## Related Work

Apart from collaborative filtering, there exist some other recommendation algorithms, which are mainly combined with other technologies or information, such as latent factor model [32], Restricted Boltzmann Machines (RBM) [33], graph-based method [34], social network analysis [35], tag information [36], matrix factorization [37], cross-domain learning [38], etc.

Researchers in Yahoo! used the latent factor model (LFM) for connecting user and item by latent factors to make a creative design scheme in the Yahoo! home page [32]. Restricted Boltzmann Machines (RBM) [33], one of the most important network structures in deep learning, was utilized to make a movie recommendation on the Netflix dataset. The visible layer of RBM is the rating matrix and hidden layer is the user features. The rating to a new query movie from a target user can be deduced by the hidden layer. A graph-based algorithm [34] was developed to recommend fantastic items to users, where the user behavior can be expressed as bipartite graph and the correlation between disjunct user nodes and item nodes can be measured by other existing links. Besides, the social network information can be used to generate a highly reliable user trust network which can be combined with user similarities to improve recommendation performance and address the cold start problem [35]. In [36], a tag system, inferring users’ similarity according to items’ tags and users’ rating records, was combined with the collaborative filtering algorithm to improve recommendation quality. In order to solve the problem of large sparse matrix and improve the running speed of recommendation algorithms, matrix factorization technologies [37] were proposed to extract user and item feature vectors from the user-item rating matrix which are used to predict ratings. Recently, cross-domain recommendation algorithms have been utilized [38], which compute the correlations in different domains and design models that exploit user preferences in a source domain to predict user preferences in a target domain.

Although some efforts have been made on asymmetric similarity recently, there still exist some deficiencies. In [39], an asymmetric similarity measure named Tversky index was proposed. In [40], the authors used an asymmetric similarity measure to distinguish users’ patterns. Besides, another asymmetric user similarity model based on matrix factorization was proposed in [31]. However, to our best knowledge, none of the above methods explicitly considers both of the preference of a user to different items and *explicit* global importance factor. In fact, the preference of users would be different even if the items they have already purchased are the same, and the global importance of all the users would be diverse even if they cause the same directed influence to a user.

In this paper, by proposing a new user-based collaborative filtering algorithm termed Asymmetric User Influence and Global Importance Values (AUI&GIV), we aim to address the above issues by designing an asymmetric user influence model and utilizing the PageRank algorithm to calculate the global importance values.

## Materials and Methods

In this paper, we propose a novel user-based collaborative filtering algorithm termed AUI&GIV (Asymmetric User Influence and Global Importance Values). In what follows, we will describe the proposed algorithm in detail, which contains three major parts, namely asymmetric user influence model, global user importance measurement and score prediction.

### Asymmetric User Influence Model

The asymmetric user influence model calculates the directed influence between two users according to the users’ purchase history. Different users give different ratings to the same item and we can infer whether the user likes the item according to the rating. In [23, 30, 36], the differences between ratings and users’ average rating are used to get the similarity between users based on the adjusted cosine. In general, if the rating given to an item is larger than a user’s average rating, it is regarded that the user likes the item. Otherwise, the user dislikes the item. An item is defined as a positive item *w.r.t.* the user who has rated it if the user likes it and the item is a negative item if the user dislikes it. In our model, we aim to get positive influences between users according to the positive items *w.r.t.* users and use the positive influence to predict the rating to un-purchased items so the negative items can be ignored. The main reason is that when a novice user wants to find a reliable expert, he is more willing to trust someone who likes the same items as him and what the novice user cares about are the items the expert likes rather than the expert dislikes. Besides, a user is more likely to trust someone with the same tastes. If an expert likes most of the items that a novice user likes, the novice user thinks that the expert shares the same items’ tastes as him and will buy the other items the expert likes. In general, an expert has more influence to a user if the expert likes more items that the user likes. That is, concerning positive items, the number of which would be much smaller than the number of all items purchased by the user, more positive items with higher ratings means that the user can make more influence to the other users, e.g., with more expertise to select high quality items. Therefore, the influence from a user to a target user should be measured according to the positive items shared by the user and the target user.

**Fig 1:**
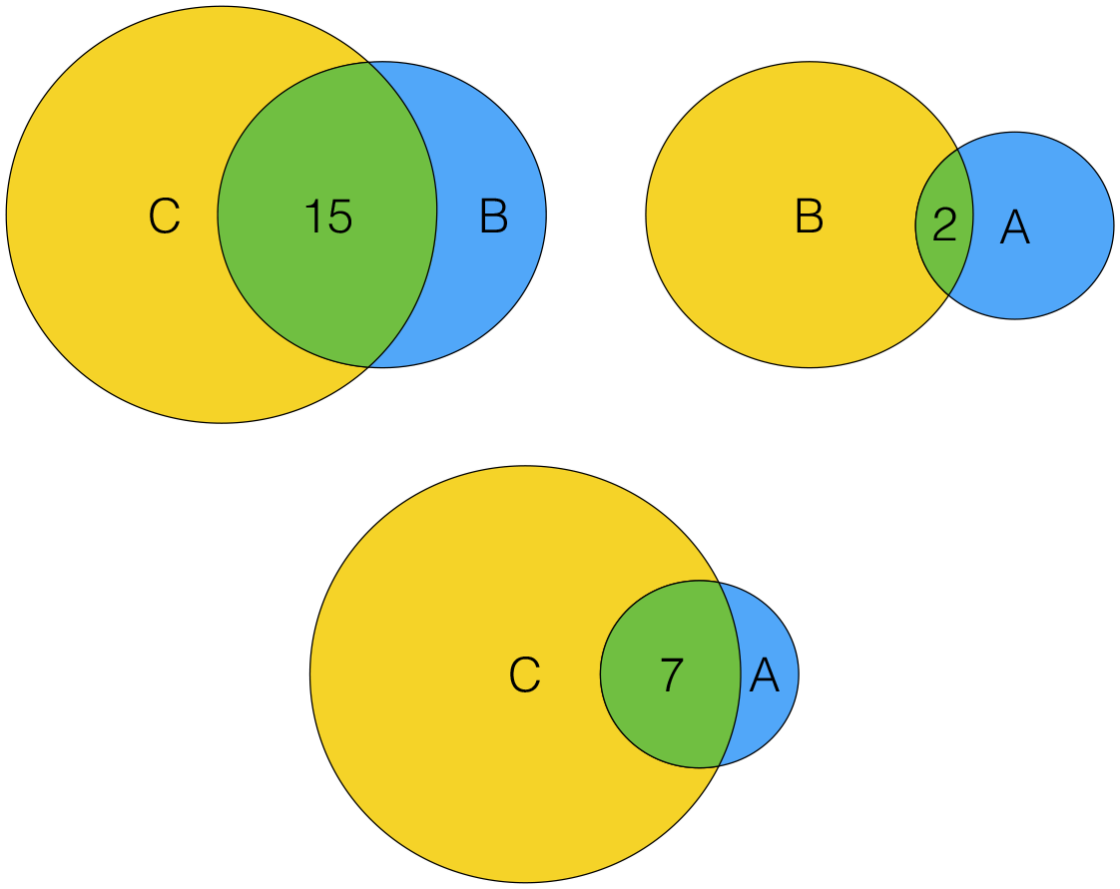
User co-positive items.

**Fig 2:**
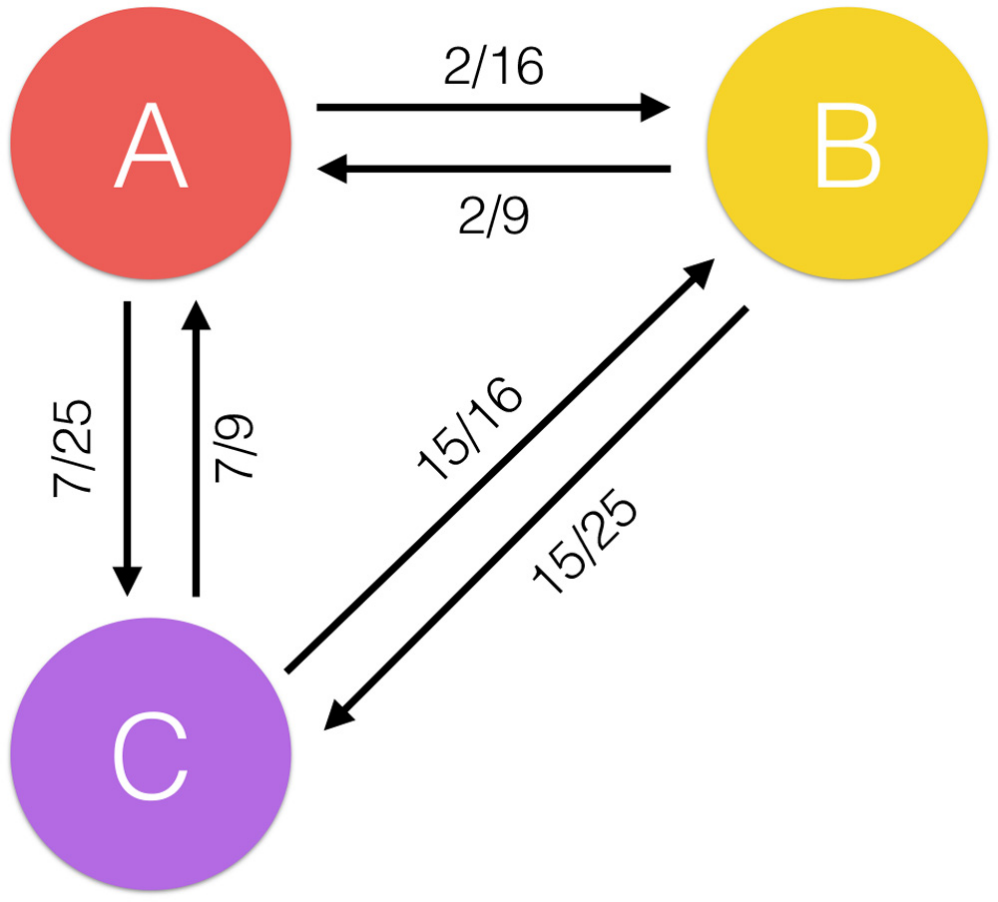
User directed influence.

For instance, as shown in Fig 1, user *A* has 9 positive items, user *B* has 16 positive items and user *C* has 25 positive items. User *B* and user *A* have 2 co-positive items, user *B* and user *C* have 15 co-positive items, and user *C* and user *A* have 7 co-positive items. The directed influence value from user *C* to user *B* is 15/16 and the directed influence value from user *A* to user *B* is 2/16. Although it may be the case that user *B* would be more selective than user *C* and therefore would not be interested in user *C*’s other positive items, yet compared with the other users (e.g. user *A*), user *C* can make more influence to user *B* in selecting high quality items, which also means that user *C* shares relatively more positive-item tastes with user *B*. Therefore, user *B* will be more likely to follow user *C*’s tastes rather than user *A*’s tastes to purchase more items. The directed influence between the three users is shown in Fig 2.

Based on the above analysis, we can derive the asymmetric user influence model as follows. Given a user-item rating matrix *R* = [*r*_*ui*_]_*m*×*n*_, which represents the entire *m* × *n* user-item rating relation, where *m* is the number of users and *n* is the number of items, each entry *r*_*ui*_ in line *u* and column *i* denotes the rating of user *u* to item *i* within a certain numerical interval that varies in different datasets. The higher the rating value *r*_*ui*_ is, the more user *u* is fond of item *i* and if user *u* has not yet rated item *i*, the value of *r*_*ui*_ is set to zero.

First of all, we need to compute the average rating ${\bar{r}}_{a}$ of user *a* to all items as follows,

$${\bar{r}}_{a}=\frac{\sum_{i=1}^{n}r_{ai}}{n_{a}}$$ (1)

where *n*_*a*_ is the number of items purchased (rated) by user *a*. Accordingly, we can select the positive items by using the average rating ${\bar{r}}_{a}$ of each user *a* to construct a boolean matrix $R^{\prime }={\left[r_{ai}^{\prime }\right]}_{m\times n}$. The element $r_{ai}^{\prime }\in \left\{0,1\right\}$ indicates the preference of user *a* to item *i*, i.e. whether user *a* likes item *i*. That is, if *r*_*ai*_ is larger than ${\bar{r}}_{a}$, $r_{ai}^{\prime }$ is equal to one which indicates that user *a* likes item *i*. Otherwise, $r_{ai}^{\prime }$ is set to zero. That is

$$r_{ai}^{\prime }=\left\{\begin{array}{cc} 1, & \text{if}\;r_{ai}\geq {\bar{r}}_{a}\\ 0, & \text{otherwise.} \end{array}\right.$$ (2)

And then, we can construct the asymmetric user influence matrix *W* according to the previous boolean matrix *R*′, which is denoted as *W* = [*w*_*uv*_]_*m*×*m*_, where *w*_*uv*_ represents the influence from user *u* to user *v*. We can use the ratio of the number of co-positive items to the number of positive items *w.r.t.* of user *v* to get the value of *w*_*uv*_ as follows,

$$w_{uv}=\frac{\sum_{i=1}^{n}r_{ui}^{\prime }\times r_{vi}^{\prime }}{\sum_{i=1}^{n}r_{vi}^{\prime }}.$$ (3)

It is obvious that the user influence matrix is an asymmetric matrix which is different from the symmetric user relationship matrix used in the traditional user-based collaborative filtering algorithm [21–23].

### Global User Importance Measurement

**Fig 3:**
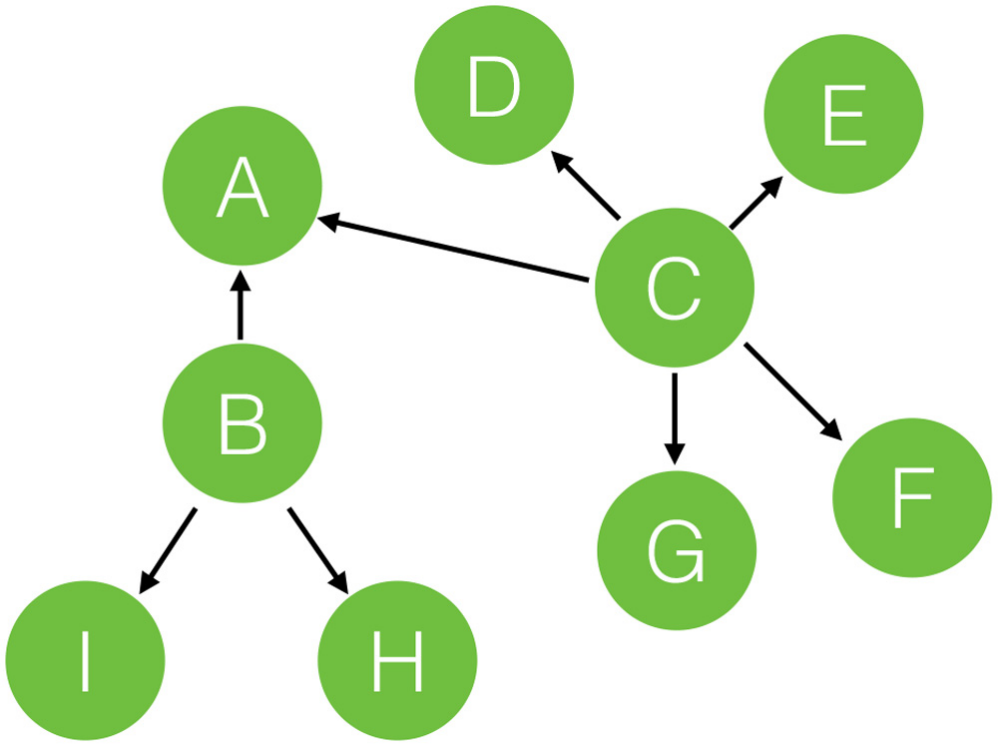
User global importance.

It is assumed that 1) the more users a user can affect, the more important the user is and 2) if there are several users who can make the same influence to the target user, the target user is more easily affected by the user with the largest importance value. For instance, if user *B* and user *C* make the same influence to user *A* and user *C* can affect more users than user *B* (i.e., larger importance value) as shown in Fig 3, then it is more likely that user *A* is affected by user *C* since user *C* is more authoritative because he can cause influence to more users.

Based this assumption, we can conclude that apart from the asymmetric user influence matrix, the global user importance value also plays a key role in improving the accuracy of recommendation.

To this end, we propose to use the PageRank algorithm to calculate the importance value of each user. Intuitively, if a user can affect more users in a group, he is a more important person who has more significant influence to all the users. So, we suppose that the more users affected by the user, the more important the user is. The in-degree of a user is the number of users who can affect him and the out-degree is the number of users this user can affect. If the influence value between user *u* and user *v* is larger than the average influence, the indicator variable $w_{uv}^{\prime }$ is equal to one and it is likely that user *u* can influence user *v*. The average influence value $\bar{w}$ and indicator variable $w_{uv}^{\prime }$ are calculated respectively as follows,

$$\bar{w}=\frac{\sum_{u=1}^{m}\sum_{v=1}^{m}w_{uv}}{\sum_{u=1}^{m}\sum_{v=1}^{m}\delta \left(w_{uv}\right)},\;\;\;w_{uv}^{\prime }=\left\{\begin{array}{cc} 1, & \text{if}\;w_{uv}\geq \bar{w}\\ 0, & \text{otherwise} \end{array}\right.$$ (4)

where *δ*(*x*) is a delta function such that *δ*(*x*) = 1 if *x* > 0 and *δ*(*x*) = 0 otherwise.

If $w_{uv}^{\prime }$ is equal to one, user *u* can affect user *v*. On the contrary, user *u* can not affect user *v* at all if $w_{uv}^{\prime }$ is equal to zero. Therefore, we can obtain the out-degree *c*_*u*_ of each user *u* by their indicator variable vector $c_{u}=\sum_{v=1}^{m}w_{uv}^{\prime }$.

We initialize all users’ PageRank value with randomly generated positive values and use the following iteration method to get the PageRank value of each user. In each iteration *t*, the PageRank value is updated as

$$pr_{u}^{t}=\frac{\left(1-d\right)}{m}+d\times \sum_{v\in in(u)}\frac{pr_{v}^{t-1}}{c_{v}}$$ (5)

where *d* ∈ [0, 1] is a damping coefficient representing a scaling factor of the contribution of other users to the user and *in*(*u*) is a collection gathering all the users who can affect user *u*. The process terminates if all the PageRank values are stable.

The PageRank algorithm is used to calculate the authority of all the pages by Google search engine, the page with higher in-degree and lower out-degree gets a higher PageRank value when the algorithm converges which reflects the page is more authoritative. We should notice that, in our algorithm, the user with lower in-degree and higher out-degree is more important. By Eq (5), the user who affects more users and is affected by less users gets a smaller PageRank value, but the smaller PageRank value reflects the more important the user is. If a user causes larger directed influence to another user, the asymmetric user influence from the user to another user is much larger. To keep things consistent, the global importance value of a more important person should be mapped to a larger importance value.

One simple strategy for addressing this issue is to apply a monotonically decreasing function [41] to adjust all users’ PageRank values.

$$pr_{u}=\frac{1}{1+\log pr_{u}}.$$ (6)

However, the importance values of most users generated by this strategy are considerably larger than their influence values. If so, when we combine the users’ influences and users’ importances to make a prediction, the latter will mistakenly overpower the former in the recommendation process. The range of the asymmetric user influence value is [0, 1]. For consistency, all the PageRank value of users should be mapped to the Interval [0, 1]. Therefore, such a simple strategy is not applicable. To this end, our algorithm utilizes another mapping function that is used in logistic regression [42], which can map all the PageRank values into the Interval [0, 1] as follows,

$$pr_{u}=\frac{1}{1+e^{-pr_{u}}}.$$ (7)

Finally, we can get the global importance values for all users, which are stored as a vector, i.e. *PR* = [*pr*_1_ … *pr_n_*].

### Score Prediction

We use the product of asymmetric user influence value and the user global importance value to make a prediction for the rating of a target user to an unrated item. Let *p*_*ai*_ denote the predicted rating of user *a* to item *i*, then the rating can be calculated as follows,

$$p_{ai}={\bar{r}}_{a}+\frac{\sum_{u\in U_{i}}w_{ua}\times pr_{u}\times \left(r_{ui}-{\bar{r}}_{u}\right)}{\sum_{u\in U_{i}}\left|w_{ua}\times pr_{u}\right|}$$ (8)

where *i* is the item that has not been rated by user *a*, *U*_*i*_ is a collection gathering all the users who have rated item i, *w*_*ua*_ is the influence value from user *u* to user *a*, and *pr*_*u*_ is the importance value of user *u*.

## Results

In this section, extensive experiments are conducted to evaluate the effectiveness of the AUI&GIV algorithm on four well-known real-world datasets including Jester, EachMovie, MovieLens and Netflix [43]. First of all, we will analyse the necessity of the two components in the proposed AUI&GIV algorithm, namely asymmetric user influence model and global user importance value. The results show that both of the two components play key roles in improving the recommendation accuracy. Then, we will analyse the sensitivity of the proposed algorithm to the damping parameter used in PageRank when computing the global user importance value, experimental results of which show that different damping factors lead to relatively stable results. Finally, we will compare our algorithm with seven existing recommendation algorithms. Comparison results have confirmed the effectiveness of the proposed algorithm. In particular, significance tests show that the proposed algorithm can significantly improve the performance of the traditional user-based CF algorithm on datasets of high rating density.

### Datasets and Evaluation Metrics

#### Datasets

In our experiments, four widely tested real-world datasets are used, namely Jester, EachMovie, MovieLens and Netflix.

**Fig 4:**
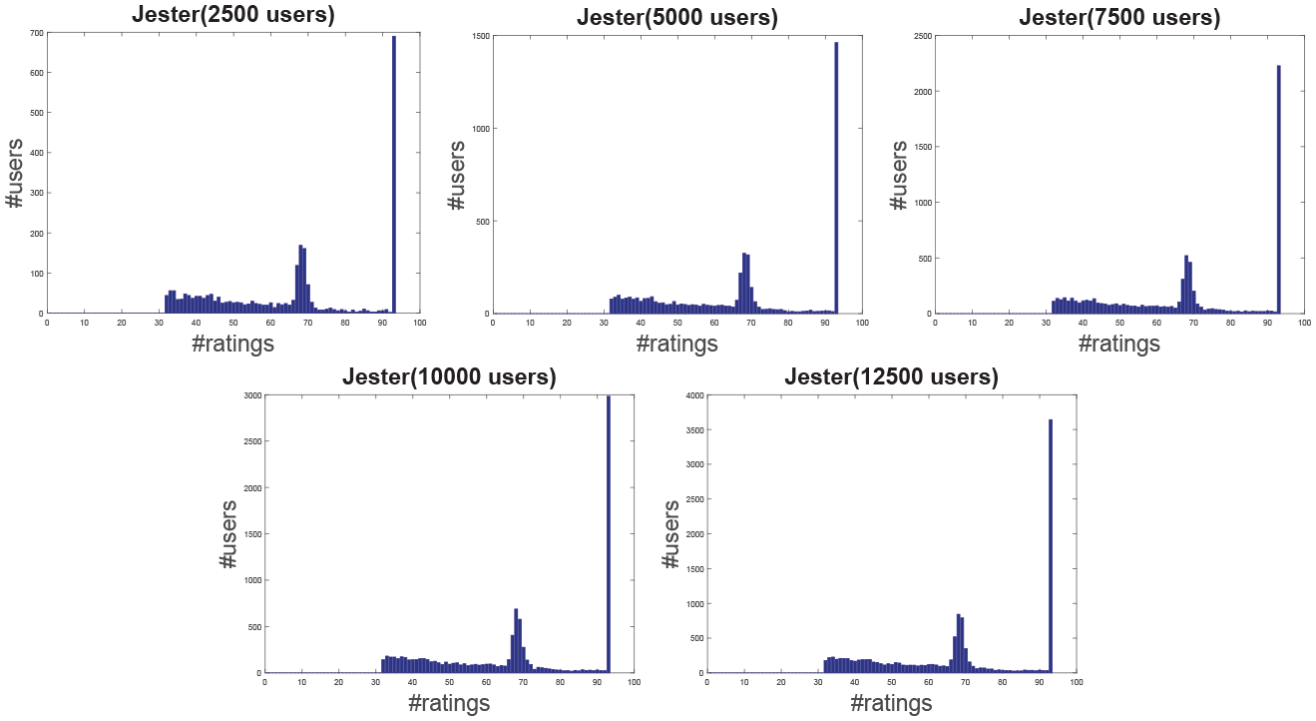
The distribution of number of ratings over users on the Jester dataset.

**Fig 5:**
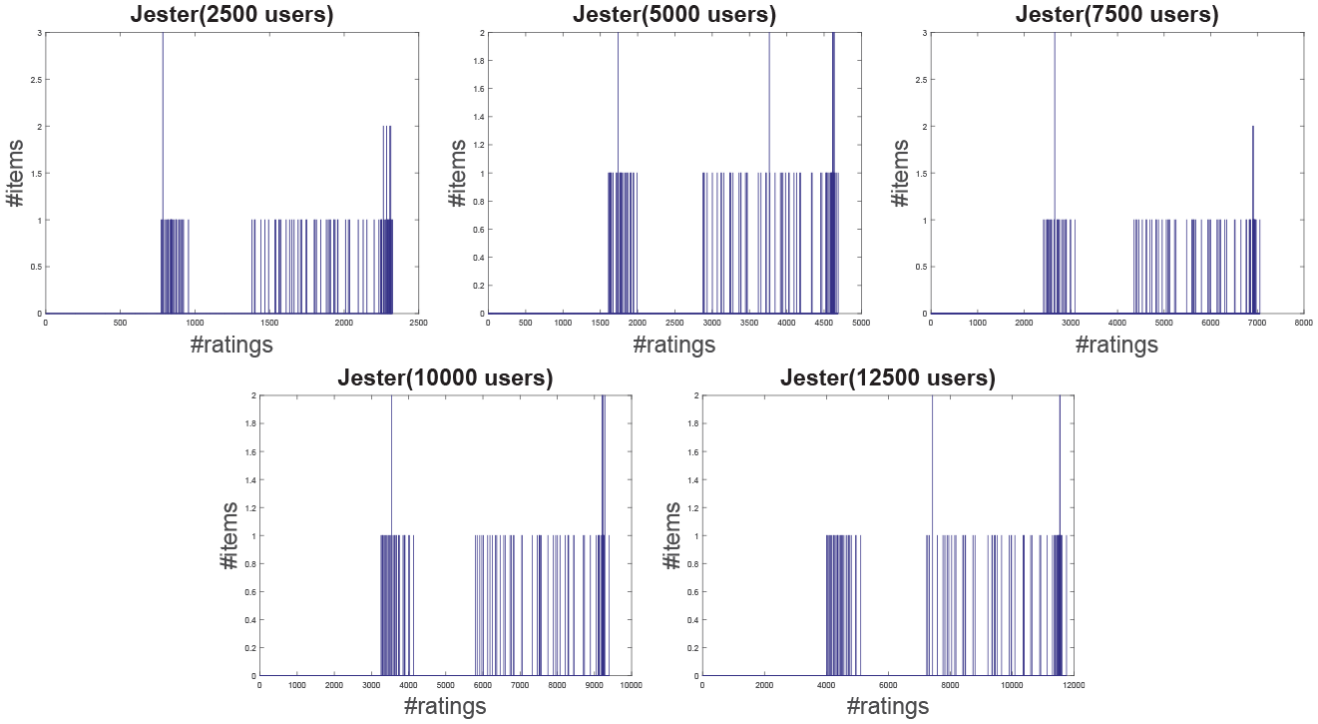
The distribution of number of ratings over item on the Jester dataset.

**Fig 6:**
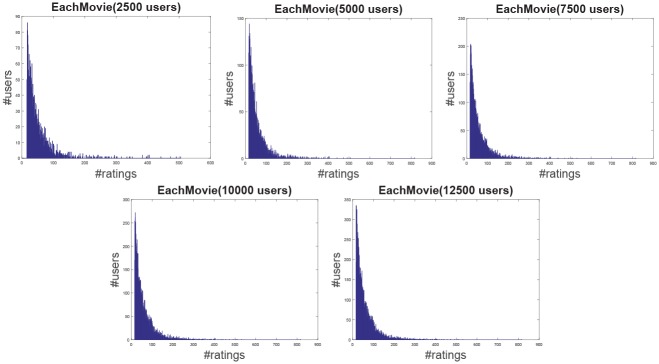
The distribution of number of ratings over users on the EachMovie dataset.

**Fig 7:**
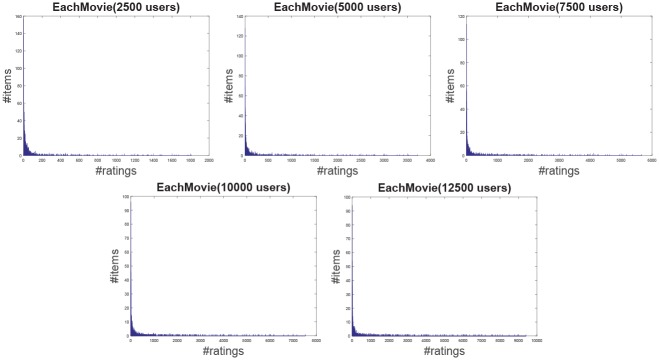
The distribution of number of ratings over items on the EachMovie dataset.

**Fig 8:**
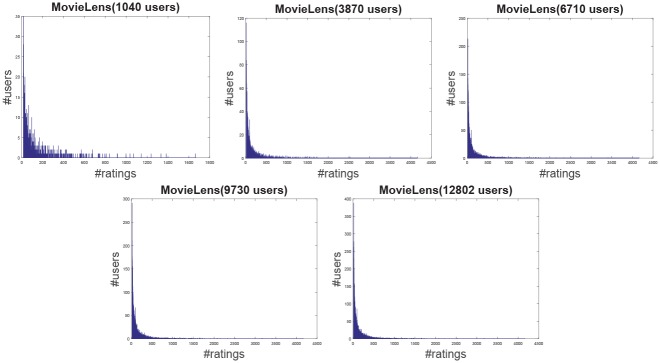
The distribution of number of ratings over users on the MovieLens dataset.

**Fig 9:**
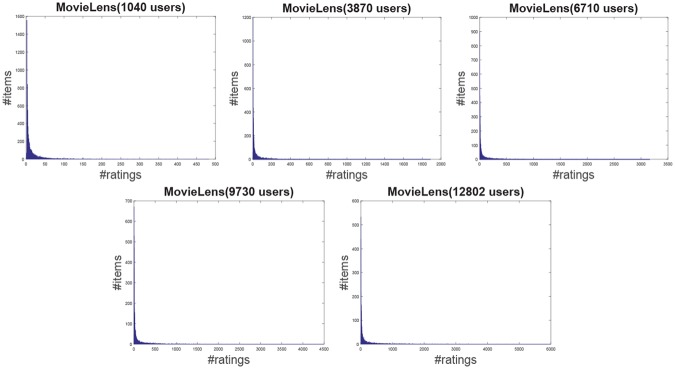
The distribution of number of ratings over items on the MovieLens dataset.

**Fig 10:**
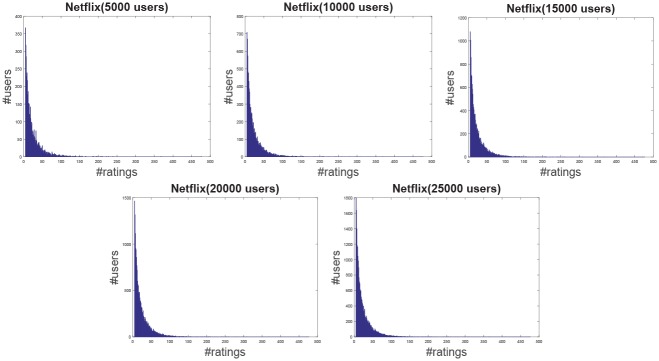
The distribution of number of ratings over users on the Netflix dataset.

**Fig 11:**
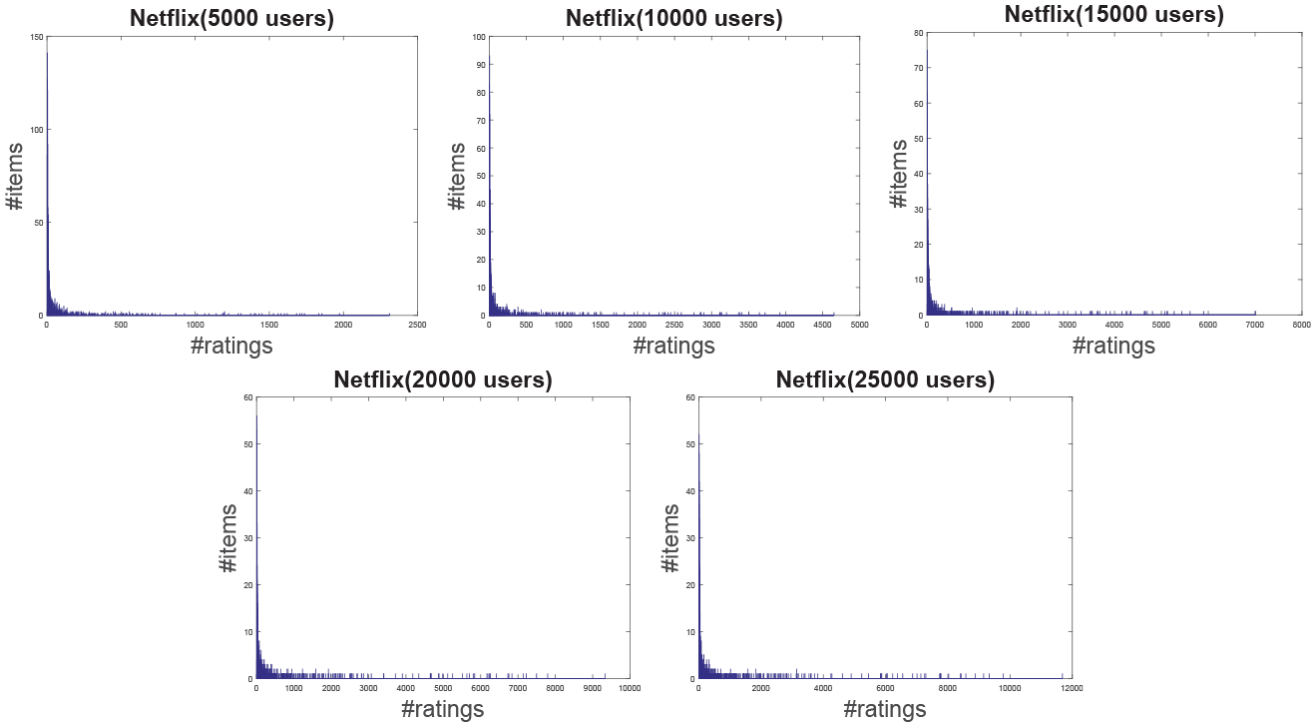
The distribution of number of ratings over items on the Netflix dataset.

- **Jester**: The Jester dataset collects 4.1 million continuous ratings (-10.00 to +10.00) of 100 jokes from 73421 users from April 1999 to May 2003 provided by University of California. There are some jokes almost all users have rated and all the jokes have a large number of rating records so the user-item rating matrix is very dense. In other words, each user has rated enough items. The original dataset contains many data files, some of which may overlap and each of which has different properties. Therefore, each data file should be used separately. Like the previous works [44], we just use one of those in our experiment which contains 12500 users and extract five sub files from the original file by random samples with different scales as shown in Table 1. The distributions of number of ratings over users and items are shown in Figs 4 and 5 respectively. In the data file, we select 90% rating records as the training data and the remaining 10% as the testing data randomly. For convenience we map the range of the rating from the interval [−10, 10] to [0, 20].
- **EachMovie**: The DEC Systems Research Center has collected 2811983 numeric ratings of 72916 users for 1628 different movies, which is called EachMovie dataset. Each rating record contains Person_ID: Number, Movie_ID: Number, Score: Number (0 to 1), Weight: Number and Modified: Date/Time, but the weight is not used in recommendation so we can ignore the value in each record. Because each rating record contains a timestamp, we group the whole dataset firstly according to Person_ID and then sort each group by the timestamp. So the rating records in each group are from the same user and our task is to predict the rating of the last movie the user watched in each group. Similar to the Jester dataset, we extract five sub datasets from the original dataset by random samples with different number of users as shown in Table 1 and the distributions of number of ratings over users and items are shown in Figs 6 and 7 respectively.
- **MovieLens**: The dataset contains 10000054 ratings (1 to 5) and 95580 tags applied to 10681 movies by 71567 users of the online movie recommender service MovieLens. All the users have rated at least 20 movies but the user-item rating matrix is still quite sparse since the number of movies is far larger than 20. Similarly, five sub datasets are extracted by selecting different users randomly from the original data file as shown in Table 1 and the distributions of number of ratings over users and items are shown in Figs 8 and 9 respectively. We conduct a 9:1 split sorted by the timestamp into training set and testing set respectively on each sub dataset, while guaranteeing that each user appears in both training set and testing set.
- **Netflix**: It’s also a famous movie dataset providing 100480507 ratings that 480189 users gave to 17770 movies from October 1998 to December 2005. The ratings are on a scale from 1 to 5 stars. Unlike the EachMovie and MovieLens datasets, there is many noise data in the dataset, i.e. some users have rated only a few movies. Therefore, preprocessing is applied to remove users who have rated only one movie so as to guarantee that each user should appear in both training and testing sets. Similarly, five sub datasets are extracted by random samples as shown in Table 1 and the distributions of number of ratings over users and items are shown in Figs 10 and 11 respectively.

**Table 1:** Datasets.

Jester	#Users	2500	5000	7500	10000	12500
	#Items	100	100	100	100	100
	#Records	176715	176715	541321	723105	901367
	Density	0.7069	0.6703	0.7218	0.7231	0.7211
EachMovie	#Users	2500	5000	7500	10000	12500
	#Items	1546	1501	1528	1535	1545
	#Records	131492	276435	432846	589139	736675
	Density	0.0340	0.0368	0.0378	0.0384	0.0381
MovieLens	#Users	1040	3870	6710	9730	12802
	#Items	6728	8925	9574	9914	10068
	#Records	126804	505818	888775	1317295	1729636
	Density	0.0191	0.0146	0.0138	0.0137	0.0134
Netflix	#Users	5000	10000	15000	20000	25000
	#Items	1500	1500	1500	1500	1500
	#Records	116064	230046	346722	463309	579081
	Density	0.0155	0.0153	0.0154	0.0154	0.0154

According to Table 1 and Figs 4 to 11, we can see that Jester is the densest dataset among the four datasets. The density here is defined as the ratio of the number of ratings (i.e. nonzero entries) to the number of entries in rating matrix (i.e. #Users×#Items). From the user’s perspective, most of the users have rated many items and many users have rated about 90% items. From the item’s perspective, most of the items have been rated by many users. By contrast, the other three datasets are quite sparse, where most of the items just receive ratings from a small amount of the users and most of users just give ratings to a few number of items. The sparsities of the MovieLens and Netflix datasets are similar and the EachMovie dataset is slightly denser than MovieLens and Netflix but still sparse.

#### Evaluation Metrics

To evaluate the quality of the recommendation algorithms, two widely used evaluation metrics are used, namely Mean Absolute Error (MAE) and Root Mean Square Error (RMSE) [36]. Assume that the collection of the predicted ratings of the target user is [*p*_1_, *p*_2_, …, *p*_*N*_] and the collection of the actual ratings of the corresponding user is [*q*_1_, *q*_2_, …, *q*_*N*_], the metrics of MAE and RMSE are computed respectively as follows,

$$MAE=\frac{1}{N}\sum_{i=1}^{N}\left|p_{i}-q_{i}\right|,\;\;\;\;RMSE=\sqrt{\frac{\sum_{i=1}^{N}{\left(p_{i}-q_{i}\right)}^{2}}{N}}$$ (9)

where *N* is number of items we are going to predict for the target user. The smaller the MAE or RMSE value is, the more accurate the prediction is. After getting the predicted ratings, we recommend items to a target user according to the predicted ratings from highest to lowest. So the algorithm generating a smaller MAE or RMSE value has a better performance.

### Component and Parameter Analysis

#### Component Analysis

The AUI&GIV algorithm has two components: asymmetric user influence model and global user importance value. In this subsection, we will demonstrate that both of the two components play key roles in improving the recommendation accuracy.

**Fig 12:**
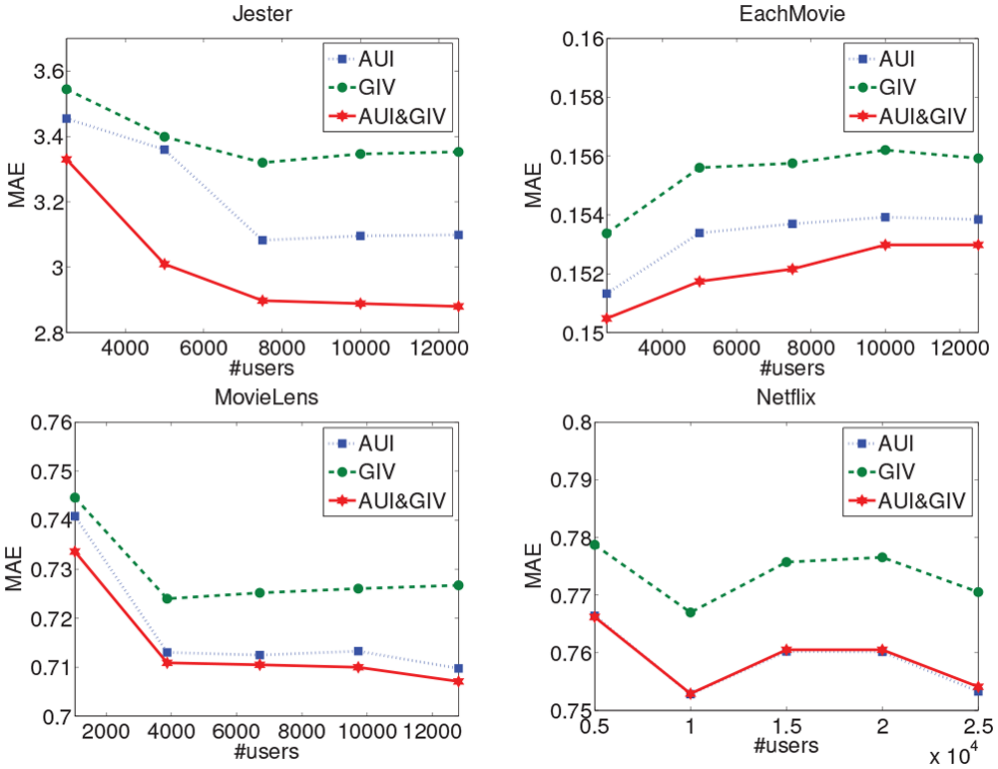
The MAE of the component analysis on the four datasets.

**Fig 13:**
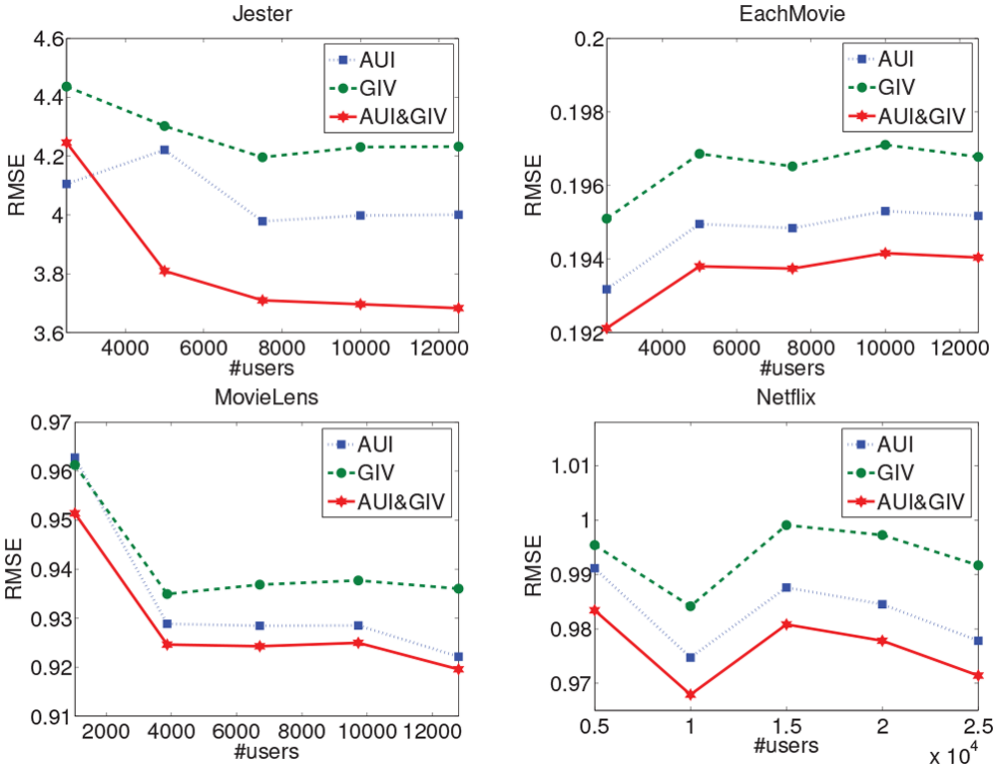
The RMSE of the component analysis on the four datasets.

To analyse the necessity of the two components, we execute each component separately and their combination on the four different datasets with different scales. As shown in Figs 12 and 13, the single AUI algorithm which just considers the asymmetric user influence outperforms the single GIV algorithm which just considers each single user’s importance value without the relation between them on all the datasets, i.e., the MAE and RMSE of the AUI algorithm are lower than those of the GIV algorithm. When we combine the two components, the algorithm generates the best results in most of cases. Especially on the Jester dataset which is dense with most of users and items having many rating records, the GIV component has a big impact so the performance of the combinatorial algorithm AUI&GIV is much better than the single AUI and GIV algorithms. On the MovieLens and Netflix datasets which are the sparsest datasets used in our experiments, the GIV algorithm just improves the performance of the AUI algorithm slightly. Although the distributions of number of ratings over items and users are similar on the three sparse datasets (i.e. MovieLens, Netflix and EachMovie), the GIV component can achieve more improvements on the EachMovie dataset because it is slightly denser than the other two sparse datasets. In general, the GIV component does not make the AUI algorithm worse but slightly better in most of cases, for both sparse and dense datasets. However, the impact of the GIV component is more obvious as the density of dataset increases. This clearly indicates that the main factor to measure the relationship between users is their asymmetric influence. We can draw the conclusion that the asymmetric influence between the users contributes more to the entire algorithm and the user’s global importance value computed by the PageRank algorithm can improve the quality of the algorithm slightly by amending each user’s final influence.

**Fig 14:**
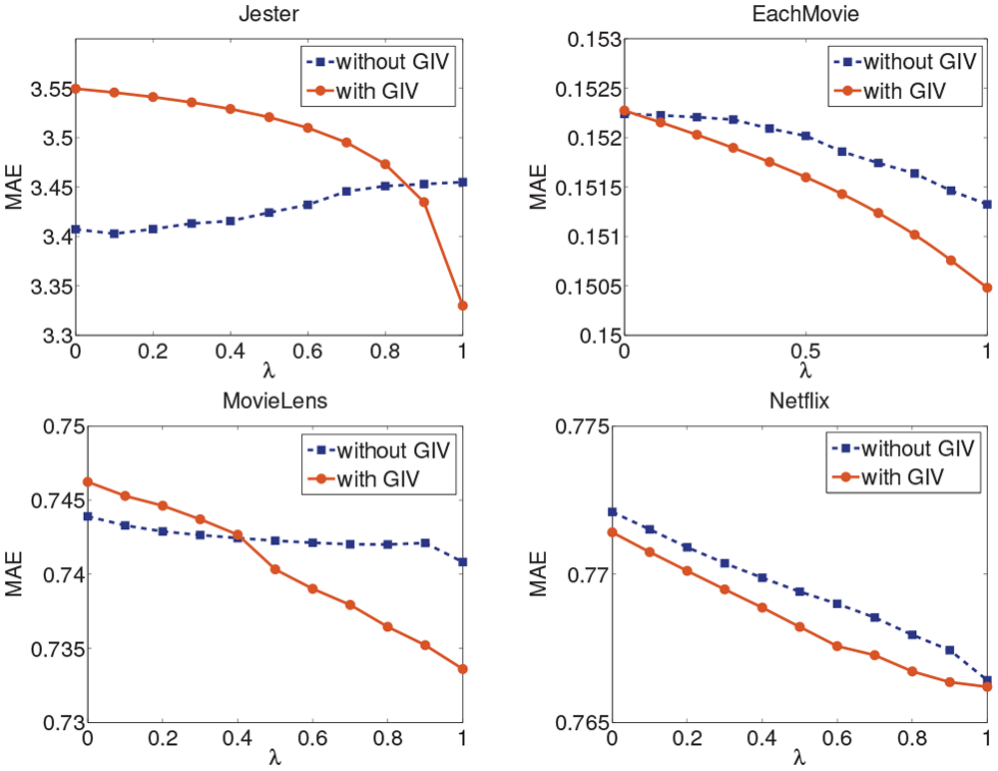
The MAE of the hybrid algorithm on the four datasets.

**Fig 15:**
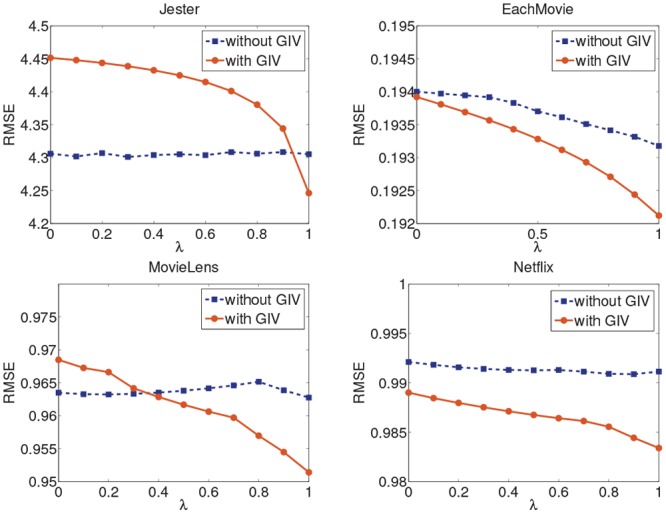
The RMSE of the hybrid algorithm on the four datasets.

We use the method mentioned in [27, 45] to get the traditional symmetric user similarity matrix *S* = [*s*_*uv*_]_*m*×*m*_ with *s*_*uv*_ representing the similarity between user *u* and user *v*

$$s_{uv}=\frac{\sum_{i\in I_{uv}}\left(r_{ui}-{\bar{r}}_{u}\right)\left(r_{vi}-{\bar{r}}_{v}\right)}{\sqrt{\sum_{i\in I_{uv}}{\left(r_{ui}-{\bar{r}}_{u}\right)}^{2}}\sqrt{\sum_{i\in I_{uv}}{\left(r_{vi}-{\bar{r}}_{v}\right)}^{2}}}$$ (10)

where *I*_*uv*_ is a collection gathering all the items rated by user *u* and user *v*. In Figs 14 and 15, we linearly combine the traditional symmetric user influence (e.g. denoted as *s*) and the asymmetrical user influence (e.g. denoted as *w*) to get a hybrid influence *s* × (1 − *λ*) + *w* × *λ* when the global user importance value is used, where *λ* is the coefficient of the user influence within the range [0, 1]. When the variable *λ* is equal to 0, the combination algorithm becomes a user-based CF recommendation algorithm, and it becomes the AUI algorithm when *λ* is equal to 1. We test the hybrid algorithm on the four datasets with smallest sizes respectively.

As shown in the two figures, the quality of the hybrid algorithm is improved if *λ* keeps increasing. However, when *λ* is small, the effect of the hybrid algorithm is worse than the algorithm without GIV. As *λ* increases, the asymmetrical user influence makes more effect in the hybrid influence and the hybrid algorithm with GIV gets a better performance. Therefore, conclusion can be drawn that the user importance values computed by the PageRank algorithm can optimize asymmetrical user relationship but it does not benefit the symmetric user relationship. This may be because the PageRank-based calculation of user importance values uses the asymmetrical user relationship, which differs from symmetric user similarity.

#### Parameter Analysis

**Fig 16:**
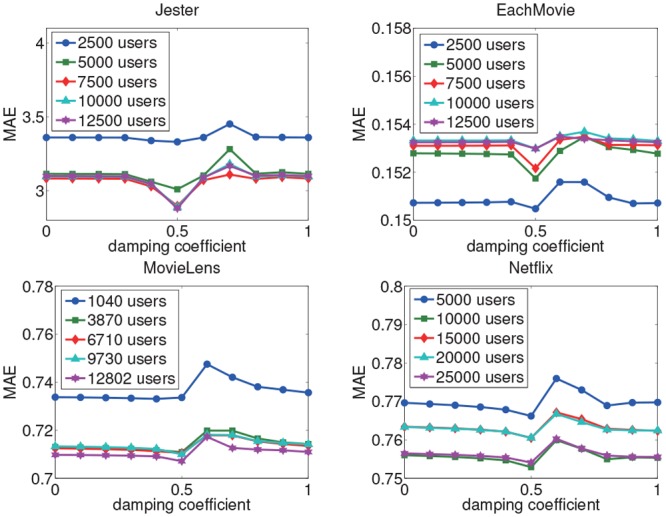
The MAE with different damping coefficient values in the PageRank algorithm.

**Fig 17:**
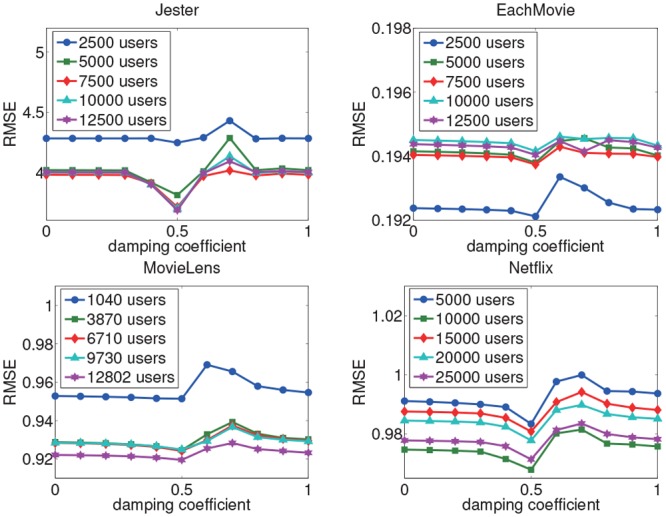
The RMSE with different damping coefficient values in the PageRank algorithm.

We also analyse the effect of various damping values on the performance of the AUI&GIV algorithm for rating prediction. The analysis is also conducted on the five subsets associated with each dataset. We vary the damping coefficient value from 0 to 1, and set the step size to 0.1. The results are shown in Figs 16 and 17. We can observe that the performance of the AUI&GIV algorithm is optimal when the damping coefficient *d* is set to 0.5 in most cases. And the performances are almost the same when *d* is equal to 0 and 1, both of which cannot match the performance with *d* = 0.5. In general, we can achieve better results when we set *d* < 0.6. According to the PageRank algorithm, when an expert has affected a user, the probability the other users affect the user is the damping coefficient. If the probability is equal to zero, which means all the users’ importance values are random values and hence is not affected by other users, in this case, the PageRank algorithm does not work very well. In other words, we can not measure the user importance values well without the probability. But the probability a user is influenced by others should not be too large according to the experiments. Therefore, in the AUI&GIV algorithm, we suggest to set the damping coefficient *d* = 0.5 to get good performance.

### Comparison Experiments

In this subsection, we present the performance comparison on the predicted ratings in terms of accuracy between our AUI&GIV algorithm and seven existing recommendation methods on the four datasets with five different scales. In our AUI&GIV algorithm, we set the damping coefficient *d* = 0.5 as analysed above. The compared methods with their experiment setting will be described briefly first, and then the comparison results will be reported.

#### Compared Methods

In comparison experiments, seven existing recommendation algorithms are compared, namely User-Based collaborative filtering with Matrix Factorization (UBMF) [46], User-Based Collaborative Filtering (UBCF) [27], Item-Based Collaborative Filtering (IBCF) [28], Slope One [47], K-Nearest Neighbor (KNN) [48], Improved Singular Value Decomposition (SVD++) [49] and MF-AMSD [31].

1. User-Based collaborative filtering with Matrix Factorization(UBMF) [46]: This algorithm is an extension of User-based CF, which not only uses matrix factorization to extract the user features from the user-item matrix but also reduces the dimension of the user vector to some small number (e.g. 2 in our experiments). Afterwards, we can compute the user similarities from the extracted user feature matrix and predict the rating in the same way as User-Based CF.
2. User-Based Collaborative Filtering (UBCF) [27]: User-based collaborative filtering computes the users’ similarities according to the users’ rating records and recommends the items that the similar users have purchased to the target user.
3. Item-Based Collaborative Filtering (IBCF) [28]: Item-based collaborative filtering measures the similarities between items and recommends the items which are the most similar to the items the target user has already bought to the target user.
4. Slope One [47]: The basic idea of the algorithm is linear regression. The first step of the algorithm is to compute the average difference of two items by their ratings. And the predicted rating to an item is produced by the items the target user has purchased and the average difference between those items and the item to be predicted.
5. K-Nearest Neighbor (KNN) [48]: Similar to IBCF, the first step of the algorithm is also to compute all items’ similarities. But it needs to filter the items’ similarities and just leave *k* most similar items for each item. By trial and error, we use the best *k* = 5 in our experiments.
6. SVD++ [49]: Single Value Decomposition is one of the matrix factorization algorithms for collaborative filtering. This algorithm finds the features of users and items by decomposing the user-item rating matrix, and uses *k* dimensional feature vectors to represent each user and each item respectively. Therefore, we can make predictions based on these factors. By introducing user biases and item biases, SVD++ is one of the improved variants which can obtain a better performance than SVD. In SVD++, the dimension of user and item feature vectors is set to 10 while the learning rate is 0.0001, and all the regularization coefficients are set to 0.1.
7. MF-AMSD [31]: The algorithm suggests an asymmetric user similarity method to distinguish the impact that the user has on his neighbor and the impact that the user receives from his neighbor, and matrix factorization is applied to the user similarity matrix to discover the similarities between users who have rated different items.

#### Comparison Results

**Fig 18:**
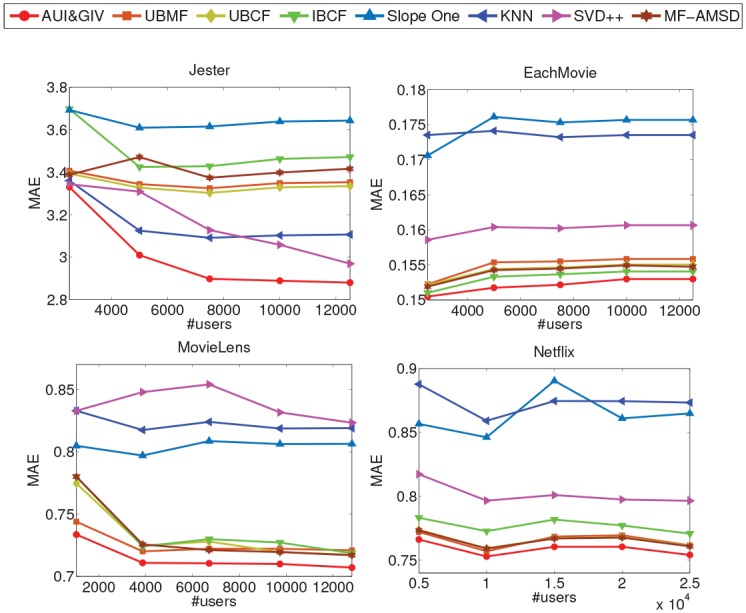
The MAE of the six recommendation algorithms on the four datasets.

**Fig 19:**
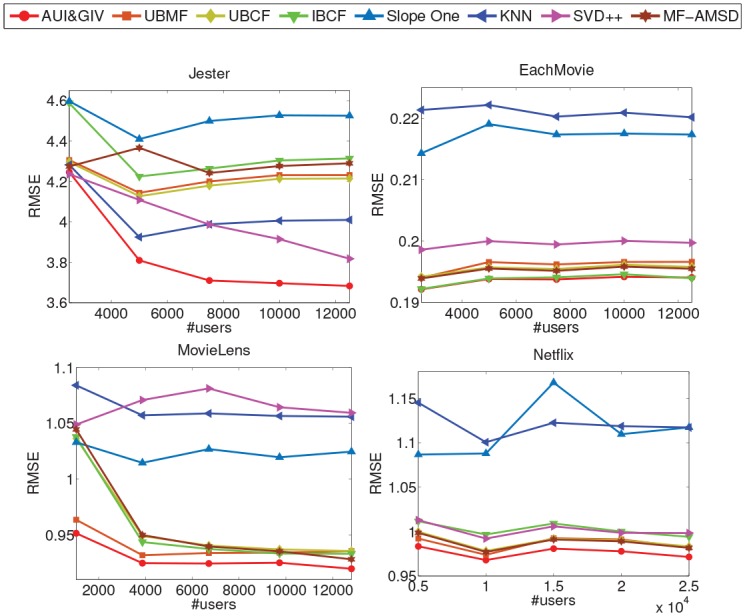
The RMSE of the six recommendation algorithms on the four datasets.

The comparison results in terms of MAE and RMSE are reported in Figs 18 and 19 respectively. The mean values (over all five subsets) of percentage gains of the proposed AUI&GIV algorithm are shown in Table 2. It’s obvious that the AUI&GIV algorithm is significantly better than all the existing recommendation algorithms. On the Jester and MovieLens datasets, the MAE and RMSE of the AUI&GIV algorithm monotonically decrease as the scale of the dataset increases.

**Table 2:** The mean values of percentage gains.

Dataset	Metrics	UBMF	UBCF	IBCF	Slope One	KNN	SVD++	MF-AMSD
Jester	MAE	10.59%	10.10%	14.23%	17.57%	5.00%	5.06%	11.96%
	RMSE	9.35%	8.99%	11.80%	15.15%	5.32%	4.62%	10.73%
EachMovie	MAE	1.86%	1.41%	0.75%	12.93%	12.39%	5.01%	1.28%
	RMSE	1.23%	0.95%	0.08%	10.84%	12.40%	2.99%	0.81%
MovieLens	MAE	1.57%	2.47%	2.87%	11.20%	13.13%	14.72%	2.43%
	RMSE	1.13%	3.09%	2.80%	9.25%	12.57%	12.75%	3.04%
Netflix	MAE	0.90%	0.92%	2.35%	12.13%	13.15%	5.35%	0.87%
	RMSE	1.01%	1.25%	2.59%	12.29%	12.88%	2.52%	1.13%

The accuracies generated by the Slope One algorithm are quite poor on the four datasets, i.e., its MAE or RMSE is quite large and the AUI&GIV algorithm can achieve the improvement from 9% to 17%. The performances of the UBCF and UBMF recommendation algorithms are almost the same, but the former is slightly more accurate than the latter because the processing of feature extraction loses some precisions. It is also obvious that the traditional UBCF recommendation algorithm is slightly better than the traditional IBCF recommendation algorithm in most cases when the user relationship matrix and item relationship matrix are both symmetric. The performance of the MF-AMSD algorithm is pretty good because it uses a more reasonable method to measure the user relationship than UBCF. In general, the performance of the UBMF, UBCF, IBCF and MF-AMSD algorithms are close to the AUI&GIV algorithm on almost all the four datasets except the Jester dataset but still not as good as AUI&GIV. The AUI&GIV algorithm can achieve 0.08% to 3.09% improvement compared with the four algorithms on the three sparse datasets. However, the percentage gains improved on the Jester dataset are as high as 10%. The KNN algorithm doesn’t work as well as our method, but is still impressive on the Jester dataset because all the users on the Jester dataset have rated a relatively large number of items which does some favor to KNN by getting rid of noise. Compared with the KNN algorithm, the AUI&GIV algorithm can make about 5% improvement on the Jester dataset but 12% on the other sparse datasets. Being sensitive to the parameter, the performances of the SVD++ algorithm vary significantly on the four datasets. The performance of SVD++ on the MovieLens dataset is the worst, and even not as good as collaborative filtering on the EachMovie and Netflix datasets, but it becomes better as the scale increases on the Jester dataset.

Therefore, the UBMF, UBCF, IBCF and MF-AMSD algorithms can perform well on the sparse datasets. On the contrary, the KNN and SVD++ algorithms can perform well on the dense dataset but poorly on sparse data. The proposed method significantly outperforms the others on the Jester dataset but slightly on the other three sparse datasets. But anyway the AUI&GIV algorithm always gets the best results on all the datasets whether sparse or not. Besides, the AUI&GIV is the only one algorithm which can perform well on both dense and sparse datasets compared with the other traditional recommendation algorithms.

#### Significance Tests

To make the above analysis scientifically more precise, significance tests will been conducted to show the effect of asymmetric user influence and global importance value on the improvement of its counterpart algorithm, i.e., user based CF (UBCF), for both dense data and sparse data. To this end, two null hypotheses are proposed respectively for dense data and sparse data as follows.

1. Null hypothesis I:
  Using asymmetric user influence and global importance value has no effect on the UBCF algorithm on dense data.
  The corresponding alternative hypothesis I is that using asymmetric user influence and global importance value does have effect on the UBCF algorithm on dense data.
2. Null hypothesis II:
  Using asymmetric user influence and global importance value has no effect on the UBCF algorithm on sparse data.
  The corresponding alternative hypothesis II is that using asymmetric user influence and global importance value does have effect on the UBCF algorithm on sparse data.

In our experiments, according to the statistics on datasets, it is clear that Jester is a typical dense dataset, i.e. of high rating density w.r.t. both users and items. On the other hand, the other three datasets, namely EachMovie, MovieLens and Netflix, are typical sparse datasets, i.e. of low rating density w.r.t. both users and items.

According to Table 2, when comparing AUI&GIV and its counterpart UBCF on dense dataset, i.e. Jester, significant improvement (10.10% in terms of MAE and 8.99% in terms of RMSE) has been achieved, which is much higher than the common significance level 5%. This significance test entails that the null hypothesis I is rejected, which means that the experimenter can now conclude that using asymmetric user influence and global importance value *does* have effect on the UBCF algorithm on dense data, i.e. the alternative hypothesis I is true.

On the other hand, when comparing AUI&GIV and its counterpart UBCF on sparse datasets, i.e. EachMovie, MovieLens and Netflix, significant improvement cannot be achieved, i.e., lower than common significance level 5%. This significance test only shows that the null hypothesis II cannot be rejected. However, in this case, we cannot conclude that the null hypothesis II is true, i.e. we cannot accept the null hypothesis II just because it is not rejected.

According to the above significance tests, conclusion can be drawn that using asymmetric user influence and global importance value does have effect on the UBCF algorithm on dense data.

## Conclusion

In this paper, we have proposed a novel user-based collaborative filtering algorithm termed AUI&GIV. Different from the traditional user based CF algorithm, the user relationship in our algorithm is asymmetric which means if a user has an impact on another user, the latter has a different impact on the former or even may not affect the former at all. So we first compute the directed influence between the users according to their positive rating records. Besides, we also consider the global importance values of the users by using the PageRank algorithm. The basic idea is that, a user would have a larger importance value if many users have been affected by the user, and the larger the importance value is, the more people would trust the user intuitively. Next, we combine the asymmetric user influence and the global importance to measure the final influence of all the users. Finally we use the user influence matrix and the user rating matrix to predict the rating of un-purchased items for all the users. Extensive experiments have been conducted on four widely-used datasets. The results have confirmed that both the two components in our method play key roles in improving recommendation accuracy, and hence the proposed method significantly outperforms the existing recommendation algorithms. Also significance tests have been conducted to show that using asymmetric user influence and global importance value does have effect on the UBCF algorithm on dense data.
